# The ghrelin-growth hormone axis preserves gluconeogenesis to maintain blood glucose levels during starvation

**DOI:** 10.1186/1753-6561-6-S3-P65

**Published:** 2012-06-01

**Authors:** Daniel P Sherbet, Tongjin Zhao, Robert L Li, Michael S Brown, Joseph L Goldstein

**Affiliations:** 1 Department of Molecular Genetics, University of Texas Southwestern Medical Center, Dallas, Texas, 75390, USA

## Background

The octanoylated peptide ghrelin stimulates growth hormone (GH) secretion during severe calorie restriction in mice, which preserves fasting blood sugar after body fat has been depleted. Genetic deletion of ghrelin-O-acyltransferase (GOAT, which octanoylates ghrelin) renders mice ghrelin-deficient and abrogates the normal rise in GH after several days of calorie restriction, resulting in profound hypoglycemia. Administration of either ghrelin or GH to GOAT knockout mice restores the ability to maintain fasting blood sugar levels during prolonged calorie restriction. The mechanism of hypoglycemia in ghrelin-deficient mice has not been described previously.

## Materials and methods

8-week-old male wild-type (WT) and GOAT knockout mice were individually caged and fed 40% of their average daily food intake at 6pm daily during the period of calorie restriction. For glucose production measurements, 4 days prior to calorie restriction each mouse was implanted with a jugular vein catheter. On day 6 of calorie restriction at 4 pm, glucose production was assessed by infusing [3-^3^H]glucose through the catheter. Blood was obtained from cut tails during the infusion and plasma was separated by centrifugation and deproteinized. The supernatant was evaporated, resuspended in water, and radioactivity was quantified with liquid scintillation counting.

## Results

GOAT and ghrelin knockout mice fail to show the normal rise in GH when subjected to fasting after several days of 60% calorie restriction. Both mutant strains become fat-depleted and develop profound hypoglycemia under these conditions. High fat feeding of GOAT knockout mice prior to calorie-restriction doubled body fat percentage and delayed onset of hypoglycemia from day 6 of calorie restriction to day 12. This was associated with a delayed decline in plasma free fatty acids and β-hydroxybutyrate. Plasma levels of lactate and pyruvate in calorie-restricted GOAT knockout mice were half the levels of WT mice. Fasting hypoglycemia in GOAT knockout mice was associated with a glucose production rate that was only 40% of the rate in WT mice. Administration of lactate, pyruvate, and alanine, which can be used as gluconeogenic precursors, restored blood glucose in GOAT knockout mice. Administration of octanoate, which cannot be used as a gluconeogenic precursor, increased glucose production to the level seen in WT mice and restored blood glucose, presumably by providing energy for gluconeogenesis.

## Conclusions

Ghrelin-mediated stimulation of GH is critical for maintaining blood glucose in fasting mice when fat stores have been depleted. GH maintains plasma gluconeogenic precursors and preserves glucose production when endogenous (fat) and exogenous (food) fuel sources are absent. These studies demonstrate that ghrelin and GH allow mice to maintain blood glucose during starvation. Elevated levels of both hormones are also seen in starved humans with anorexia nervosa or kwashiorkor, suggesting that the ghrelin-GH system prolongs life during starvation in both mouse and man.

